# Apple Peel Flavonoid Fraction 4 Suppresses Breast Cancer Cell Growth by Cytostatic and Cytotoxic Mechanisms

**DOI:** 10.3390/molecules24183335

**Published:** 2019-09-13

**Authors:** Chao-Yu Loung, Wasundara Fernando, H.P. Vasantha Rupasinghe, David W. Hoskin

**Affiliations:** 1 Department of Pathology, Faculty of Medicine, Dalhousie University, Halifax, NS B3H 4R2, Canada; joe.loung@dal.ca (C.-Y.L.); wasufer@dal.ca (W.F.);; 2 Department of Plant, Food, and Environmental Sciences, Faculty of Agriculture, Dalhousie University, Truro, NS B2N 5E3, Canada; 3 Department of Pathology, Department of Microbiology and Immunology, Department of Surgery, Faculty of Medicine, Dalhousie University, Halifax, NS B3H 4R2, Canada

**Keywords:** apoptosis, breast cancer, cell cycle, flavonoids, reactive oxygen species, tumor suppression

## Abstract

Many dietary flavonoids possess anti-cancer activities. Here, the effect of apple peel flavonoid fraction 4 (AF4) on the growth of triple-negative (MDA-MB-231, MDA-MB-468), estrogen receptor-positive (MCF-7), and HER2-positive (SKBR3) breast cancer cells was determined and compared with the effect of AF4 on normal mammary epithelial cells and dermal fibroblasts. AF4 inhibited breast cancer cell growth in monolayer cultures, as well as the growth of MCF-7 spheroids, without substantially affecting the viability of non-malignant cells. A sub-cytotoxic concentration of AF4 suppressed the proliferation of MDA-MB-231 cells by inhibiting passage through the G_0_/G_1_ phase of the cell cycle. AF4-treated MDA-MB-231 cells also exhibited reduced in vitro migration and invasion, and decreased Akt (protein kinase B) signaling. Higher concentrations of AF4 were selectively cytotoxic for MDA-MB-231 cells. AF4 cytotoxicity was associated with the intracellular accumulation of reactive oxygen species. Importantly, intratumoral administration of AF4 suppressed the growth of MDA-MB-231 xenografts in non-obese diabetic severe combined immunodeficient (NOD-SCID) female mice. The selective cytotoxicity of AF4 for breast cancer cells, combined with the capacity of sub-cytotoxic AF4 to inhibit breast cancer cell proliferation, migration, and invasion suggests that flavonoid-rich AF4 (and its constituents) has potential as a natural therapeutic agent for breast cancer treatment.

## 1. Introduction

The regular consumption of fruits and vegetables that are rich in flavonoids and other bioactive molecules is associated with a reduced risk of developing various types of cancers [1,2,3,4,5]. It is unlikely that any single natural source-derived compound is responsible for this beneficial effect since multiple bioactive molecules in flavonoid-rich foods are likely to act in a synergistic fashion. Numerous studies on phytochemicals as potential anti-cancer agents have found that these natural compounds affect many different signaling pathways involved in cancer development and progression [6,7]. It is hoped that further research on the anti-cancer properties of phytochemicals will lead to the development of plant-based therapeutics for the prevention or treatment of cancer. 

Apples, which are a common source of dietary flavonoids, have been widely investigated for their disease-fighting properties [8,9,10,11]. Apple peel flavonoid fraction 4 (AF4) is a flavonoid-rich ethanolic extract of the Northern Spy apple cultivar [12]. AF4 contains a number of polyphenolic compounds, including flavonols, anthocyanins, dihydrochalcones, phenolic acids, and flavan-3-ols. Quercetin glycosides (quercetin-3-*O*-galactoside, quercetin-3-*O*-rutinoside, quercetin-3-*O*-glucoside and quercetin-3-*O*-rhamnoside) comprise approximately 70% of the phenolic content of AF4. Previous studies have established the neuroprotective and anti-inflammatory properties of AF4 in different mouse models [12,13]. AF4 also inhibits the growth of hepatocellular carcinoma (HepG2) cells by causing cell cycle arrest and apoptosis, as well as acting as a topoisomerase toxicant [14].

Breast cancer is the most common cancer among North American women and the second highest cause of cancer-related deaths [15]. The poor prognosis of metastatic breast cancer mandates the development of novel treatment strategies. The impact of AF4 treatment on breast cancer cells has not yet been determined. In the current study we used in vitro and in vivo approaches to elucidate the selective cytotoxic, anti-proliferative, anti-migratory and tumor suppressor effects of AF4 on breast cancer cells. 

## 2. Results

### 2.1. Apple Peel Flavonoid Fraction 4 (AF4) Selectively Inhibits the Growth of Breast Cancer Cells

**Figure 1:**
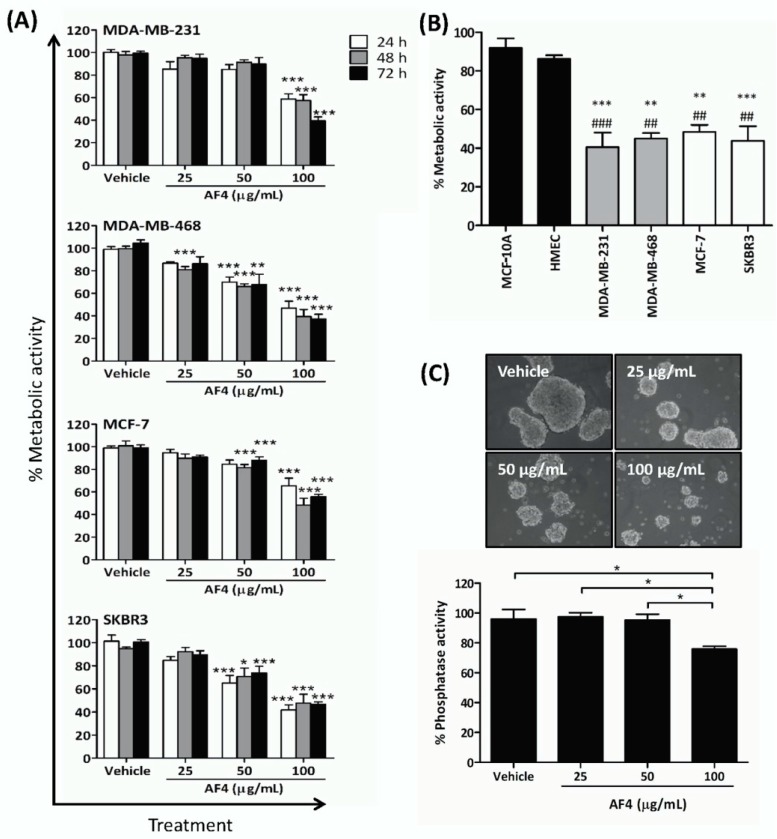
Apple peel flavonoid fraction 4 (AF4) is selectively cytotoxic to breast cancer cells. (**A**) MDA-MB-231, MDA-MB-468, MCF-7 and SKBR3 cells were cultured in the absence or presence of the indicated concentrations of AF4 for the indicated times. The % metabolic activity of AF4-treated cells was determined using an MTT (3-(4,5-dimethythiazol-2-yl)-2,5-diphenyltetrazolium bromide) assay. Data are expressed as mean ± standard error of the mean (SEM). (**B**) The % metabolic activity of AF4-treated (100 µg/mL) breast cancer cells was compared to MCF-10A cells (#) and HMECs (*) cells. Data from MTT assays are expressed as mean ± SEM. (**C**) MCF-7 spheroids were cultured for 72 h in the absence or presence of the indicated concentrations of AF4. Relative cell number of viable cells was determined by acid phosphatase assay. Data are shown as mean ± SEM. (A–C) Statistical analysis of 3 independent experiments was performed using analysis of variance (ANOVA) and Tukey’s multiple comparisons test; * *p* < 0.05, ** and ^##^ *p* < 0.01, *** and ^###^ *p* < 0.001.

Exposure to AF4 reduced the number of viable triple-negative (MDA-MB-231, MDA-MB-468), estrogen receptor-positive (MCF-7), and HER2 receptor-positive (SKBR3) breast cancer cells in monolayer cultures in a dose- and time-dependent manner, as indicated by reduced cellular metabolic activity measured by 3-(4,5-dimethythiazol-2-yl)-2,5-diphenyltetrazolium bromide (MTT) assays (Figure 1A). Sensitivity to AF4 (100 µg/mL) at 72 h was as follows: MDA-MB-231 (39% ± 3% viable) and MDA-MB-468 (38% ± 3% viable) > SKBR3 (48% ± 2% viable) > MCF-7 (56% ± 2% viable). AF4 was selective for breast cancer cells since a concentration of AF4 (100 µg/mL) that was cytotoxic for breast cancer cells had little effect on the viability of nonmalignant human mammary epithelial cells (HMEC) and MCF-10A mammary epithelial cells (Figure 1B). In comparison to quercetin, AF4 was a less potent inhibitor of breast cancer cell growth but had greater selectivity (Figure S1). MCF-7 spheroids that were grown in the presence of AF4 (100 µg/mL) were smaller than vehicle-treated control spheroids (Figure 1C). In addition, numerous floating cells with morphology characteristic of dead or dying cells were present in AF4-treated cultures, and there was a significant decrease in the number of viable MCF-7 cells, as indicated by reduced phosphatase activity, within spheroids treated with AF4 (100 µg/mL) relative to vehicle-treated controls. Therefore, AF4 inhibited breast cancer cell growth in both 2-dimensional and 3-dimensional culture systems. Subsequent experiments focused on the effect of AF4 on MDA-MB-231 cells as this breast cancer cell line was most sensitive to AF4 and readily forms tumors in immune-deficient mice.

### 2.2. AF4 Suppresses the Proliferation of MDA-MB-231 Cells

**Figure 2:**
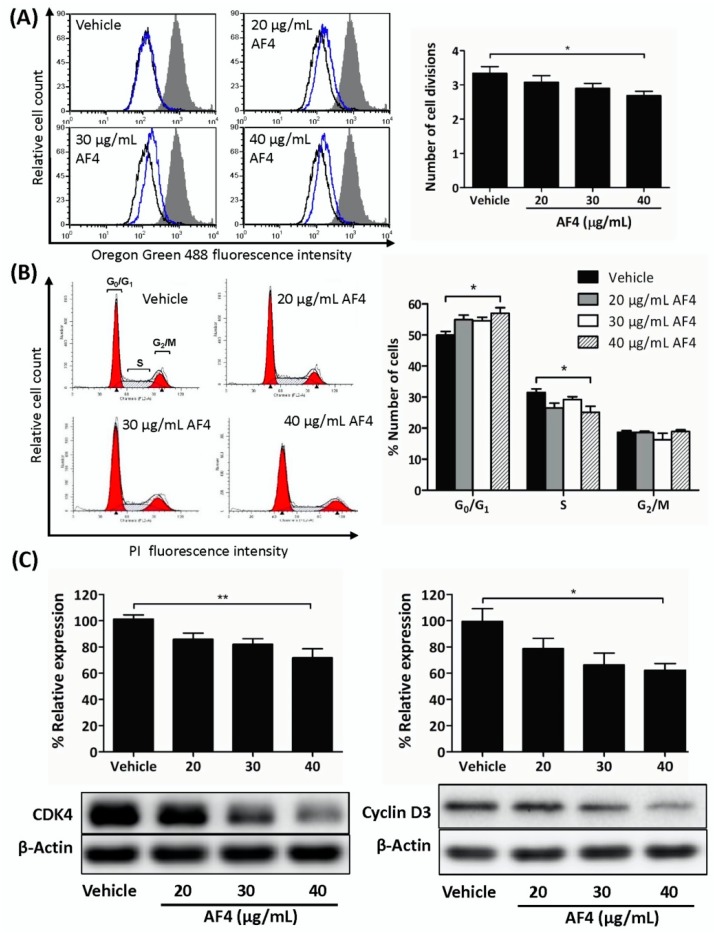
AF4 inhibits breast cancer cell proliferation. (**A**) MDA-MB-231 cells were stained with Oregon Green 488 dye and then cultured for 72 h in the absence or presence of the indicated concentrations of AF4. Fluorescence was measured by flow cytometry. Data are shown as representative histograms (filled peak, non-proliferating cells; black peak, vehicle; blue peak, AF4) and mean number of cell divisions ± SEM. (**B**) MDA-MB-231 cells were cultured for 72 h in the absence or presence of the indicated concentrations of AF4. Cells were stained with propidium iodide (PI) and cell cycle analysis was performed by flow cytometry. Data are shown as representative histograms and mean % number of cells ± SEM in each phase of the cell cycle. (**C**) MDA-MB-231 cells were cultured for 24 h in the absence or presence of the indicated concentrations of AF4. The relative expression of CDK4 and cyclin D3 was determined using Western blot analysis. Equal protein loading was confirmed by probing for β-actin. Data shown are representative blots and mean % relative expression ± SEM. (A–C) Statistical analysis of 3 independent experiments was performed using ANOVA and Tukey’s multiple comparisons test; * *p* < 0.05, ** *p* < 0.01.

Next, we determined whether a non-cytotoxic concentration of AF4 could impact breast cancer cell growth. As shown in Figure 2, flow cytometric analysis of Oregon Green 488-stained MDA-MB-231 cells that were treated with a sub-cytotoxic dose of AF4 (40 µg/mL) revealed a significant reduction in the number of cell divisions (Figure 2A). In addition, cell cycle analysis showed that AF4-treated MDA-MB-231 cells accumulated in the G_0_/G_1_ phase of the cell cycle, with a corresponding reduction in the number of MDA-MB-231 cells in the S phase of the cell cycle (Figure 2B). The same effect was observed in AF4-treated MDA-MB-468 cells (Figure S2). Consistent with an AF4-induced partial block at G_0_/G_1_, there was reduced expression of CDK4 and cyclin D3 in AF4-treated MDA-MB-231 cells (Figure 2C). 

### 2.3. AF4 Inhibits the Migration and Invasion of MDA-MB-231 Cells

**Figure 3:**
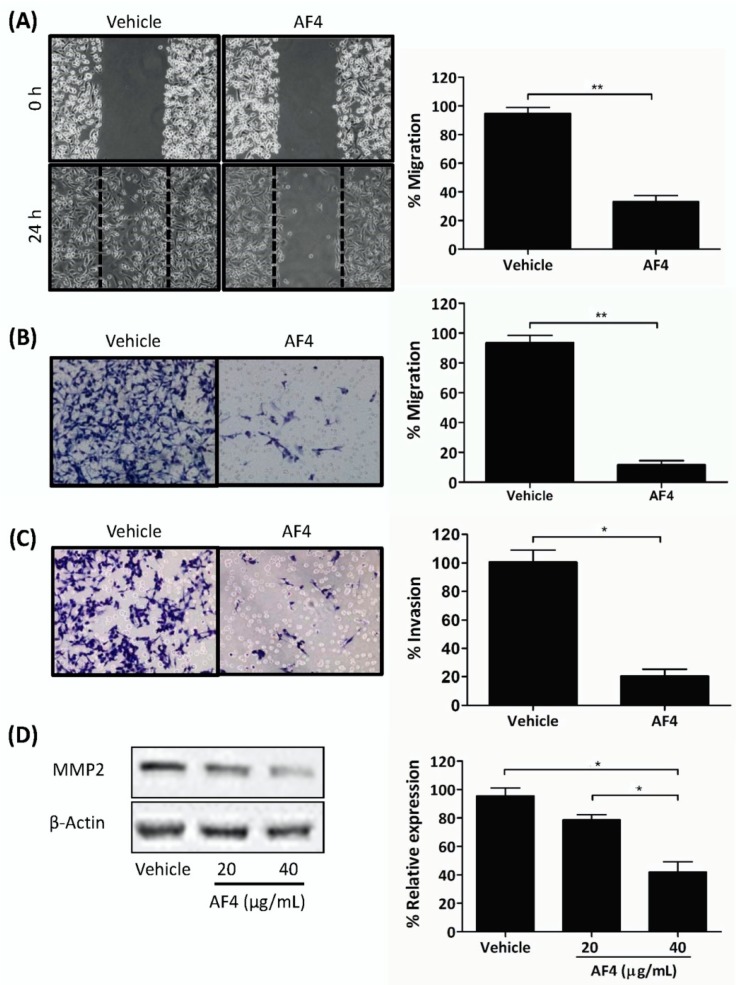
AF4 inhibits breast cancer cell motility and invasion. (**A**) Mitomycin C-treated MDA-MB-231 cells were cultured in wells containing cell culture inserts, which were removed at 0 h. After 24 h culture in the absence or presence of 20 µg/mL AF4 cultures were photographed. Representative images and mean % migration ± SEM of 3 independent experiments are shown. (**B**) Serum-starved MDA-MB-231 cells were treated with 20 µg/mL AF4 for 24 h. Mean % migration ± SEM through an 8 µm porous membrane and (**C**) mean % invasion ± SEM through a fibronectin-coated 8 µm porous membrane were determined as described in the Methods. (**D**) MDA-MB-231 cells were cultured for 24 h in the absence or presence of the indicated concentrations of AF4. Relative expression of MMP2 was determined using Western blot analysis. Equal protein loading was confirmed by probing for β-actin expression. Data shown are representative blots and mean % relative expression ± SEM. Statistical analysis of 3 independent experiments was performed using (A–C) Student’s *t*-test or (D) ANOVA and Tukey’s multiple comparisons test; * *p* < 0.01, ** *p* < 0.001.

Gap closure and trans-well migration assays were used to determine the effect of sub-cytotoxic AF4 on the migration and invasion capacity of MDA-MB-231 cells. These experiments used AF4 at a final concentration of 20 µg/mL in order to ensure that there would be no AF4-associated cytotoxic activity. As shown in Figure 3, a sub-cytotoxic concentration of AF4 (20 µg/mL) inhibited the migration of MDA-MB-231 cells by 65% in gap closure assays and by 87% in trans-well migration assays (Figure 3A and B, respectively). In addition, the invasion of MDA-MB-231 cells through a fibronectin-coated porous membrane was reduced by 80% in the presence of sub-cytotoxic AF4 (Figure 3C). Expression of invasion-promoting matrix metalloproteinase 2 (MMP2) was also significantly reduced when MDA-MB-231 cells were cultured in the presence of sub-cytotoxic AF4 (Figure 3D).

### 2.4. AF4-Induced Apoptosis Is Associated with Oxidative Stress

**Figure 4:**
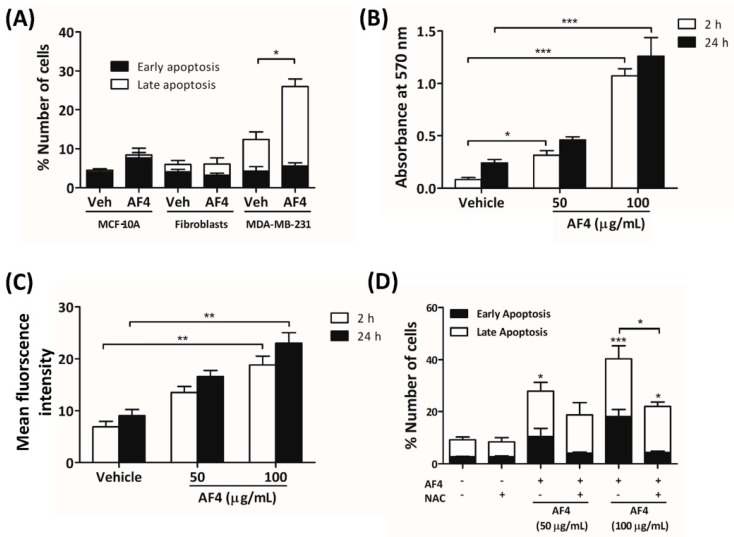
AF4-induced apoptosis of breast cancer cells is reactive oxygen species (ROS)-dependent. (**A**) MCF-10A cells, fibroblasts and MDA-MB-231 cells were cultured for 24 h in the absence or presence of 100 µg/mL AF4, and then stained with Annexin-V-488 and propidium iodide (PI) for flow cytometric analysis. Data are shown as mean % cell number ± SEM of 3 independent experiments. (**B**) MDA-MB-231 cells were cultured in the absence or presence of the indicated concentrations of AF4 for 2 h or 24 h and relative ROS in cultures was determined by Amplex Red assay. Data are shown as mean absorbance at 570 nm ± SEM of 3 independent experiments. (**C**) CM-H_2_DCFDA-stained MDA-MB-231 cells were cultured in the absence or presence of the indicated concentrations of AF4 for 2 h or 24 h and the relative amount of intracellular ROS was determined by fluorescence at 529 nm. Data are shown as mean fluorescence intensity ± SEM of 3 independent experiments. (**D**) MDA-MB-231 cells were cultured for 24 h in the absence or presence of 100 µg/mL AF4 without or with 5 mM N-acetyl cysteine (NAC), and then stained with Annexin-V-488 and PI for flow cytometric analysis. Data are shown as mean % cell number ± SEM of 3 independent experiments. (A–D) Statistical analysis was performed using ANOVA and Tukey’s multiple comparisons test; * *p* < 0.05, ** *p* < 0.01, *** *p* < 0.001.

To determine the mechanism by which AF4 killed breast cancer cells, MDA-MB-231 cells were stained with Annexin V-488 and propidium iodide (PI) prior to culture for 24 h in the presence of a cytotoxic concentration of AF4 (100 µg/mL). As shown in Figure 4A, flow cytometric analysis revealed that a high concentration of AF4 caused MDA-MB-231 cells to die by apoptosis (Figure 4A). In contrast, neither MCF-10A epithelial cells nor dermal fibroblasts were sensitive to AF4, suggesting a selective cytotoxic effect on neoplastic cells. As shown in Figure 4B,C, treatment of MDA-MB-231 cells with an apoptosis-inducing concentration of AF4 resulted in the generation of reactive oxygen species (ROS), as indicated by Amplex Red assays and flow cytometric analysis of cells stained with the ROS-sensitive dye 5-(and-6)-chloromethyl-2′,7′-dichlorodihydrofluorescein diacetate, acetyl ester (CM-H_2_DCFDA). MCF-10A cells that were cultured in the presence of AF4 (100 µg/mL) also exhibited increased levels of intracellular ROS (Figure S3). Oxidative stress was at least in part responsible for the cytotoxic action of AF4 since apoptosis of AF4-treated MDA-MB-231 cells was reduced in the presence of the antioxidant N-acetyl cysteine (NAC). In comparison to MDA-MB-231 cells, non-malignant MCF-10A epithelial cells were relatively resistant to oxidative stress (Figure S4).

### 2.5. AF4 Inhibits Akt Signaling

**Figure 5:**
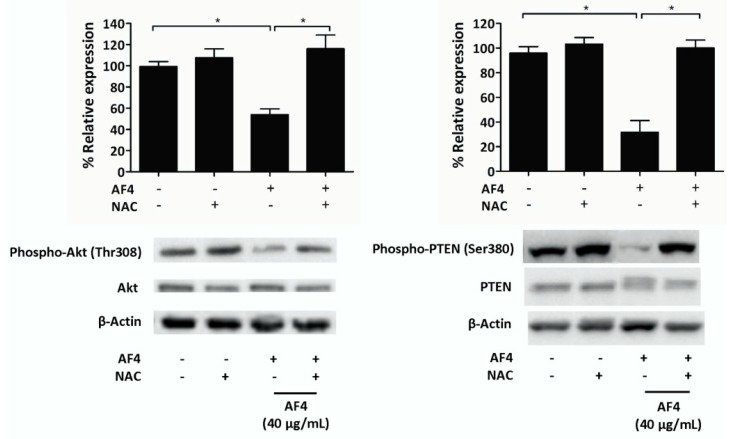
AF4 inhibits Akt and phosphatase and tensin homolog (PTEN) phosphorylation in breast cancer cells. MDA-MB-231 cells were cultured for 24 h in the absence or presence of 40 µg/mL AF4 without or with 5 mM NAC. Relative expression of phospho-Akt (Thr308), total Akt, phospho-PTEN (Ser380), and total PTEN was determined using Western blot analysis. Equal protein loading was confirmed by probing for β-actin expression. Data are shown as representative blots and mean % relative expression ± SEM of 3 independent experiments. Statistical analysis was performed using ANOVA and Tukey’s multiple comparisons test; * *p* < 0.05.

To determine a possible mechanism to account for the growth inhibitory effect of AF4, we examined the impact of AF4 on Akt (protein kinase B) signaling in MDA-MB-231 cells. A sub-cytotoxic concentration of AF4 (40 µg/mL) was used in these experiments in order to obtain sufficient protein for western blot analysis. Figure 5 shows that AF4 inhibited the phosphorylation-induced activation of Akt at Thr308. AF4 also suppressed Ser380 phosphorylation of phosphatase and tensin homolog (PTEN), which is the upstream inhibitor of Akt. Both PTEN and Akt phosphorylation were restored when AF4-treated MDA-MB-231 cells were cultured in the presence of the antioxidant NAC. The importance of the Akt signaling pathway for the growth and survival of MDA-MB-231 cells was confirmed using 2 different Akt inhibitors (MK2206 and SC66). Figure S5 shows that the percentage of apoptotic MDA-MB-231 cells increased significantly when Akt activation was inhibited.

### 2.6. AF4 Suppresses Growth of MDA-MB-231 Xenografts

**Figure 6:**
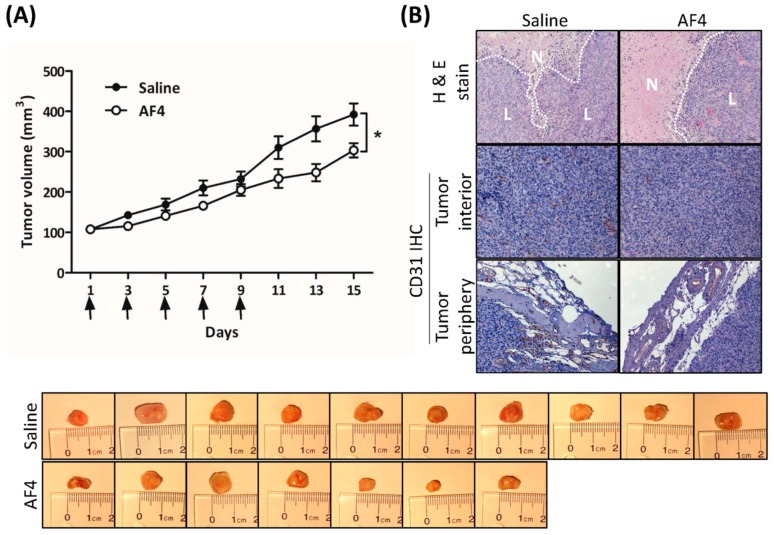
AF4 suppresses MDA-MB-231 xenograft growth. MDA-MB-231 cells were xenografted into the left flank of non-obese diabetic severe combined immunodeficient (NOD-SCID) female mice and AF4 (0.5 mg/kg) or saline was injected directly into the resulting tumors every second day (day 1, 3, 5, 7 and 9, indicated by arrows) for 9 days. The control group consisted of 10 animals and the AF4 treatment group was 7 animals. (**A**) Mean tumor volume ± SEM was determined every second day. Statistical analysis was determined by Student’s *t*-test; * *p* < 0.05. Excised tumors from each treatment group at day 15 are shown. (**B**) At day 15 mice were euthanized and tumors were excised, fixed, and sectioned for staining with hematoxylin and eosin (H&E) and detection of CD31 expression by immunohistochemistry (IHC). Representative sections are shown; N denotes areas of necrosis and L denotes live cells.

The effect of AF4 on in vivo tumor growth was determined by intratumoral administration of AF4 (0.5 mg/kg) to MDA-MB-231 xenografts grown in female non-obese diabetic severe combined immunodeficient (NOD-SCID) mice. Dosage that was predicted to suppress MDA-MB-231 tumor growth was based on the total concentration of quercetin, quercetin glycosides, catechin, epicatechin, cyanidin-3-*O*-galactoside, phloridzin, chlorogenic acid and cafeic acid within AF4. Consistent with our in vitro findings regarding the anti-proliferative and cytotoxic activities of AF4, Figure 6A shows that treatment with AF4 significantly slowed the growth of MDA-MB-231 xenografts. Examination of tumor sections stained with hematoxylin and eosin revealed larger areas of necrosis in AF4-treated tumors (Figure 6B). Moreover, in comparison to saline-treated tumors, expression of the endothelial cell marker CD31 was reduced in the interior and periphery of AF4-treated tumors. No adverse effects were noted in AF4-treated mice, including no significant difference in average body weight between the treatment groups at day 15 (saline-treated group, 24.5 ± 0.6 g; AF4-treated group, 26.3 ± 0.5 g; *p* > 0.05 by Student’s *t*-test). 

## 3. Discussion

Dietary phytochemicals have received unique attention in the search for novel and safer cancer treatment options in light of widely documented findings that many of these natural sourced compounds kill cancer cells but are relatively non-toxic to healthy cells [14,16,17,18,19]. In this study, we used MTT assays to show that AF4 suppressed the growth of triple-negative (MDA-MB-231 and MDA-MB-468), estrogen receptor-positive (MCF-7) and HER2 receptor-positive (SKBR3) breast cancer cells but had little effect on the growth of non-malignant mammary epithelial cells (HMECs and MCF-10A), suggesting a pronounced selectivity for neoplastic cells. In contrast, quercetin, which is a minor component of AF4 [12], exhibited less selectivity for breast cancer cells.

MDA-MB-231 cells that were exposed to a low concentration of AF4 tended to accumulate at G_0_/G_1_, likely as a result of reduced expression of cyclin D3 and CDK4 that promote gene expression needed for G_1_ progression [20]. AF4-treated MDA-MB-468 cells also arrested at G_0_/G_1_. This effect of AF4 on 2 different breast cancer cell lines differed from the G_2_/M cell cycle arrest seen in cultures of AF4-treated HepG2 hepatocarcinoma cells [14], suggesting that the anti-proliferative activity of AF4 may be cell type-dependent. Treatment with a higher concentration of AF4 induced MDA-MB-231 cells to undergo apoptosis; however, the viability of non-malignant fibroblasts and MCF-10A mammary epithelial cells was not affected. AF4-induced apoptosis of breast cancer cells was consistent with an earlier report of apoptotic death of liver cancer cells following treatment with AF4 [14]. We demonstrate here, for the first time, that AF4-induced cytotoxicity was at least partially due to the accumulation of intracellular ROS because the antioxidant NAC protected MDA-MB-231 cells from the cytotoxic effect of AF4. In contrast, quercetin-induced apoptosis of MDA-MB-231 cells is reported to be ROS-independent [21], which argues against a major role for quercetin in the cytotoxic effect of AF4. Interestingly, non-malignant MCF-10A cells also showed increased levels of intracellular ROS following AF4 treatment, even though these cells were refractory to the cytotoxic effect of AF4. Multiple mechanisms exist to either prevent oxidative stress or manage the negative consequences of this condition; however, excessive production and accumulation of ROS can overwhelm these defenses [22]. In line with evidence that neoplastic cells are more sensitive to ROS than are their non-malignant counterparts [23], we found that MDA-MB-231 breast cancer cells were more sensitive to ROS than non-malignant MCF-10A mammary epithelial cells. The selective effect of AF4 on breast cancer cells may involve the capacity of healthy cells to defend against endogenous ROS [24]. Although certain components of AF4 possess anti-oxidant capabilities [25], the ability of unfractionated AF4 to protect against oxidative stress has not yet been demonstrated. In any case, a number of natural source antioxidants, at high doses, are capable of causing ROS production in cancer cells [26,27,28]. 

Importantly, AF4 inhibited the growth of MCF-7 breast cancer spheroids that, in comparison to monolayer cultures of breast cancer cells, more closely resemble the 3-dimensional structure of solid tumors [29]. In addition, sub-cytotoxic AF4 interfered with the migration of MDA-MB-231 cells in gap closure and trans-well cell migration assays, as well as inhibiting invasion of MDA-MB-231 cells through a fibronectin-coated membrane and suppressing the expression of MMP2. These findings suggest that AF4 may be able to interfere with breast cancer metastasis since tumor cell locomotion and the ability to degrade extracellular matrix components via the synthesis of proteolytic enzymes such as MMP2 play essential roles in the metastatic process [30]. 

Decreased phosphorylation of Akt in the presence of AF4 may account for its anti-proliferative and cytotoxic effects on MDA-MB-231 cells since the growth, survival, and metabolism of breast cancer cells involves activation of Akt [31]. The important role played by the Akt signaling pathway in the growth and survival of MDA-MB-231 cells was confirmed by the observation that Akt inhibition resulted in apoptosis of MDA-MB-231 cells. Phosphorylation of PTEN, which is a tumor suppressor protein that downregulates Akt signaling [32], was downregulated in AF4-treated MDA-MB-2312 cells. Decreased activation of inhibitory PTEN is consistent with the finding that AF4 did not completely block Akt activation. AF4-induced ROS production is likely to be involved in the effects of AF4 on Akt and PTEN since ROS can directly inhibit Akt via the phosphorylation of thiol groups within the protein, whereas phosphatases such as PTEN are deactivated by ROS [33]. Restoration of Akt and PTEN phosphorylation to control levels when NAC was added to cultures of AF4-treated MDA-MB-231 cells confirmed the involvement of AF4-induced ROS in modulation of the Akt signaling pathway.

Intratumoral administration of AF4 suppressed the growth of MDA-MB-231 xenografts in immune-deficient mice, most likely due to the combination of anti-proliferative and cytotoxic effects of AF4 that were revealed by our in vitro studies. In this regard, AF4-treated tumors contained larger areas of necrosis relative to saline-treated tumors. In addition, decreased expression of the endothelial cell marker CD31 in the periphery and interior of AF-4 treated tumors suggested that AF4 may inhibit angiogenesis. Notably, AF4 did not cause any distress or adverse side effects such as weight loss in treated animals. Injection of AF4 directly into the tumor was employed to eliminate potential issues of AF4 bioavailability following oral dosing. Therefore, it will be important in future studies to assess the effectiveness of oral or intraperitoneal administration of AF4 to tumor-bearing mice. Delivery of an optimal concentration of AF4 to the tumor microenvironment via the oral or intraperitoneal route will likely require a nanoparticle delivery vehicle of the type that has been reported to greatly enhance the bioavailability of other bioactive phytochemicals such as curcumin [34,35]. In this regard, nanoparticles have also been employed to deliver plant extracts with anticancer properties to various types of cancer cells [36]. Identification of the components of AF4 that are responsible for its anticancer activity could allow for the development of nanoparticles containing one or more pure compounds with greater bioactivity relative to the AF4 extract; however, it is important to note that such an approach risks the loss of any potential synergy between major and minor components of AF4. Nevertheless, demonstration of AF4-mediated in vivo tumor suppressor activity, as well as reduced proliferation/survival and motility/invasion of MDA-MB-231 triple-negative breast cancer cells following AF4 treatment, suggests that AF4 and its bioactive components warrant further investigation as potential selective natural-source agents for the treatment of triple-negative breast cancer. 

## 4. Materials and Methods 

### 4.1. Reagents 

AF4 was extracted from the peels of the apple cultivar Northern Spy, as previously described [12]. AF4 in ethanol was filter-sterilized and stored at −80 °C. Prior to use in this study, ethanol was evaporated under nitrogen gas and the AF4 residue was dissolved in sterile pyrogen-free water and aliquots of the resulting AF4 stock (10 mg/mL) were stored at −20 °C. Amplex Red reagent, Annexin-V-488, Dulbecco’s modified Eagle’s medium (DMEM), DMEM/F12, horse serum, fetal calf serum, CM-H_2_DCFDA, and PI were from Life Technologies Inc. (Burlington, ON, Canada). Hydroxyethyl piperazineethanesulfonic acid (HEPES)- and bicarbonate-buffered mammary epithelial cell medium, mammary epithelial cell growth supplement, penicillin/streptomycin solution and poly-L-lysine were purchased from ScienCell Research Laboratories Inc. (Carlsbad, CA, USA). DMEM, phenol red-free DMEM, recombinant human insulin, hydrocortisone, phosphatase substrate, Triton X-100, mitomycin C, NAC, MTT, quercetin, MK2206, and SC66 were from Sigma-Aldrich (Oakville, ON, Canada). Paraformaldehyde was from Bioshop Canada Inc. (Burlington, ON, Canada). Fibroblast cell growth medium and supplements were from Lonza Inc. (Walkersville, MD, USA). DNase-free RNase A was from Qiagen Inc. (Mississauga, ON, Canada). Recombinant epidermal growth factor and basic fibroblast growth factor were from PeproTech (Rocky Hill, NJ). Diff-Quik staining kit was from Siemens Healthcare Diagnostics (Los Angeles, CA, USA). Rodent M block, anti-rabbit horse 6 radish peroxidase (HRP)-polymer and HRP/DAB detection system were from Biocare Medical (Markham, ON, Canada). HRP-conjugated anti-β-actin monoclonal antibody (Ab), anti-cyclin D3 monoclonal Ab, anti-CDK4 rabbit Ab, anti-phospho-PTEN (Ser380) monoclonal Ab, anti-PTEN monoclonal Ab, anti-Akt monoclonal Ab, and anti-phospho-Akt (Thr308) monoclonal Ab were from Cell Signaling Technology (Beverly, MA, USA). Anti-MMP2 Ab and anti-CD31 Ab were from Abcam Inc. (Toronto, ON, Canada). HRP-conjugated-goat anti-mouse IgG Ab and HRP-conjugated-donkey anti-rabbit IgG Ab were from Santa Cruz Biotechnology (Santa Cruz, CA, USA).

### 4.2. Cell Culture 

The MDA-MB-231 breast cancer cell line was provided by Dr. S. Drover (Memorial University of Newfoundland, St. John’s, NL, Canada). MDA-MB-468, MCF-7, and SKBR3 breast cancer cell lines were from Dr. P. Lee, Dr. K. Goralski and Dr. G. Dellaire, respectively (Dalhousie University, Halifax, NS, Canada). Breast cancer cell lines were authenticated by short tandem repeat analysis conducted by ATCC (Manassas, VA, USA). All breast cancer cell lines were cultured in DMEM supplemented with 10% heat-inactivated fetal calf serum, 5 mM HEPES buffer (pH 7.4), 2 mM l-glutamine, 100 U/mL penicillin, and 100 µg/mL streptomycin, and were maintained at 37 °C in a humidified incubator supplied with 10% CO_2_. HMECs from ScienCell Research Laboratories Inc. were grown in serum-free, HEPES- and bicarbonate-buffered mammary epithelial cell medium supplemented with 1% mammary epithelial cell growth supplement and 1% penicillin/streptomycin solution, and maintained for a maximum of seven passages at 37 °C in a humidified incubator supplied with 5% CO_2_. The MCF-10A normal mammary epithelial cell line was from Dr. P. Marcato (Dalhousie University, Halifax, NS, Canada). MCF-10A cells were cultured in DMEM/F12 supplemented with 10% heat-inactivated horse serum, 10 µg/mL human insulin, 20 µg/mL EGF, 0.5 µg/mL hydrocortisone, 100 U/mL penicillin, and 100 µg/mL streptomycin. MDF-10A cultures were maintained at 37 °C in a humidified incubator supplied with 10% CO_2_. Human dermal fibroblasts from Lonza Inc. (Walkersville, MD, USA) were maintained as per the supplier’s instructions.

### 4.3. MTT (3-(4,5-Dimethythiazol-2-yl)-2,5-Diphenyltetrazolium Bromide) Assay for Cell Viability

Cells were plated in quadruplicate into 96-well flat-bottom plates at a density of 5 × 10^3^ cells/well and cultured in the absence or presence of the indicated concentrations of AF4 for desired time. At the end of culture, MTT was added to each well to a final concentration of 0.5 μg/mL. Plates were then incubated for 2 h at 37 °C, after which supernatant was removed and formazan crystals in each well were solubilized in 100 μL of dimethyl sulfoxide (DMSO). Absorbance was measured at 570 nm using an Asys Expert Microplate Reader (Biochrom Ltd., Cambridge, UK). Results are expressed as % metabolic activity relative to the medium control.

### 4.4. Acid Phosphatase Assay of Spheroid Growth

MCF-7 cells in F12 medium containing 20 ng/mL basic fibroblast growth factor, 20 ng/mL epidermal growth factor, 100 U/mL penicillin, 100 μg/mL streptomycin and B27 serum-free supplement were cultured for 48 h in ultra-low adherent cell culture plates and then treated with the indicated concentrations of AF4 or vehicle for 72 h. Spheroids were photographed, washed with phosphate-buffered saline, resuspended in 100 µL acid phosphatase assay solution (0.1 M sodium acetate at pH 5.5, 0.1% Triton-X-100, 4 mg/mL phosphatase substrate) and incubated for 2 h at 37 °C in the dark. The reaction was stopped by adding 25 μL 1 N NaOH to each well. Absorbance was measured at 405 nm using an Asys Expert Microplate Reader and % acid phosphatase activity was determined.

### 4.5. Flow Cytometric Cell Proliferation Assay

MDA-MB-231 cell cultures were synchronized by serum starvation for 20 h and then seeded into 6-well plates and allowed to form monolayers. Cells were stained with 1.25 μM Oregon Green 488 dye in serum-free DMEM and a sample of stained cells was retained for use as a non-proliferative control. The remaining cells were cultured for 72 h in the absence or presence of the indicated concentrations of AF4. Cellular fluorescence was then measured using a FACSCalibur instrument (BD Bioscience, Mississauga, ON, Canada). The fluorescence of control and AF4-treated cells was compared to that of the non-proliferative control and the number of cell divisions (n) was calculated as follows: MCF_baseline_ = (2^n^)(MCF_sample_); where n denotes the number of cell divisions and MCF denotes mean channel fluorescence.

### 4.6. Cell-Cycle Analysis

MDA-MB-231 and MBA-MB-468 cell cultures were synchronized by serum starvation for 20 h and then cultured for 72 h in the absence or presence of the indicated concentrations of AF4. Cells were then collected, washed with ice-cold phosphate-buffered saline, and ice-cold 70% ethanol was added drop-wise to the cells under constant agitation. Cells were then stored at −20°C for 24 h, washed, and resuspended in phosphate-buffered saline containing 0.02 mg/mL PI, 0.1% Triton X-100, and 0.2 mg/mL DNase-free RNase A. After incubation for 30 min at room temperature in the dark, cellular fluorescence was determined using a FACSCalibur instrument. Data were analyzed using ModFitLT V2.0 software (Becton Dickson, CA, US).

### 4.7. Gap-Closure Assay

MDA-MB-231 cells were seeded at a density of 1 × 10^4^ cells/mL into wells containing culture inserts. After 18 h of culture, cells were treated with 10 μg/mL of mitomycin C in serum-free DMEM for 2 h at 37 °C to prevent cell division. After 12 h, cells were washed with complete DMEM and cultured in the absence or presence of 20 µg/mL AF4. At 0 h and 24 h, culture inserts were removed and the gaps were photographed. 

### 4.8. Trans-Well Migration/Invasion Assay

MDA-MB-231 cells were treated with 20 µg/mL AF4 for 24 h and serum-starved for 6 h, after which 5 × 10^4^ cells in serum-free DMEM were placed into wells of the upper chamber of the trans-well cell migration apparatus. The bottom chamber wells contained DMEM plus 10% fetal calf serum as a chemoattractant. Migration of cells through an 8 μm porous membrane (uncoated or coated with fibronectin) was detected by Diff-Quik staining. Migrated cells were imaged under a light microscope.

### 4.9. Flow Cytometric Measurement of Apoptosis

MDA-MB-231 cells were seeded at a density of 1 × 10^5^ cells/well into 6-well plates and cultured in the absence or presence of the indicated concentrations of AF4 without or with 5 mM NAC for the desired time. In separate experiments, MDA-MB-231 cells were cultured for 48 h in the absence or presence of the Akt inhibitors MK2206 and SC66. At the end of culture cells were harvested, washed and stained with Annexin-V-488 and PI (1 μg/mL) for 15 min at room temperature. Flow cytometric analysis was performed using a FACSCalibur instrument. 

### 4.10. Reactive Oxygen Species (ROS) Measurements

Measurement of ROS by Amplex Red assay was performed in 96-well flat-bottom plates containing quadruplicate cultures of MDA-MB-231 cells without or with AF4 at 50 μg/mL. In the dark, 100 μL of master mix containing 25 μM Amplex Red reagent and 0.005 U/mL HRP in phenol red-free cDMEM was added to each culture. After incubation at 37 °C for 2 or 24 h, absorbance was measured at 570 nm with an Asys Expert Microplate Reader. Measurement of ROS by fluorescence was performed by staining quadruplicate cultures of MDA-MB-231 cells or MCF-10A cells with 5 μM CM-H_2_DCFDA in serum- and phenol-red free DMEM. Cells were then cultured for 2 or 24 h in the absence or presence of the indicated concentrations of AF4 in phenol-red free DMEM containing 1% fetal calf serum. Fluorescence at 529 nm was measured with a Spectramax M2 Microplate Reader (Molecular Devices, San Jose, CA, USA).

### 4.11. Western Blot Analysis

MDA-MB-231 cells were cultured in the absence or presence of the indicated concentrations of AF4 for 24 h, and then placed in ice-cold lysis buffer (50 mM Tris at pH 7.5, 150 mM NaCl, 50 mM disodium hydrogen phosphate, 0.25% sodium deoxycholate, 0.1% Nonidet P-40, 100 μM Na_3_VO_4_, 10 mM NaF, 5 mM ethylenediaminetetraacetic acid and 5 mM ethylene glycol tetraacetic acid) containing freshly added protease inhibitors (1 mM phenylmethylsulfonylfluoride, 10 μg/mL aprotinin, 5 μg/mL leupeptin, 10 μM phenylarsine oxide, 1 mM dithiothreitol and 5 μg/mL pepstatin). After 15 min, cell lysates were cleared by centrifugation and the protein concentration was determined by Bradford assay. Equal amounts of protein (20 μg) were loaded into 12% or 15% sodium dodecyl sulfate polyacrylamide gels. Separated proteins were transferred onto nitrocellulose membranes, which were then blocked by 1 h incubation in 5% non-fat milk or 5% bovine serum albumin in Tween-Tris buffered saline (TBS) solution (0.25 M Tris at pH 7.5, 150 mM NaCl, 0.2% Tween-20). Blots were probed overnight at 4 °C with an optimal concentration of primary Ab, and then washed thoroughly with Tween-TBS and probed with HRP-conjugated donkey anti-rabbit IgG Ab or anti-mouse IgG Ab, as appropriate, for 1 h at room temperature. Even protein loading was confirmed by probing the blots with HRP-conjugated rabbit anti-β actin Ab. Proteins of interest were visualized by chemiluminescence.

### 4.12. Xenograft Breast Cancer Model

Six to eight week-old female NOD-SCID mice were purchased from Charles River Canada (Lasalle, QC, Canada) and housed under sterile conditions and fed a sterilized rodent diet and water supplied ad libitum. Pathogen-free MDA-MB-231 cells (5 × 10^7^) were implanted by subcutaneous injection into the right hind flank. Starting two weeks after xenografting, tumor sizes and body weights were recorded every other day until the last day (day 15) of the experiment. Tumor volume was calculated according to the equation, (L × P^2^)/2 where L is tumor length and P is perpendicular to tumor length. Intratumoral injection of AF4 commenced once the tumors reached a volume of 100 mm^3^ (recorded as day 1). A total of 5 intratumoral injections of AF4 at 0.5 mg/kg in 20 µL saline (7 mice) or saline alone (10 mice) were administered every other day for 9 days. Mice were monitored for an additional 6 days and their tumor sizes and body weights were recorded. Mice were euthanized at day 15, after which the tumors were excised and photographed. Tumors were fixed in buffered formalin, embedded in paraffin and cut into 5 µm thick sections. Tumor sections were mounted onto glass slides and stained with hematoxylin and eosin for detection of necrotic and live cells. Immunohistochemistry was performed to detect CD31 expression. Ethics approval for animal use was obtained from the Dalhousie University Committee on Laboratory Animals, and was in accordance with Canadian Council on Animal Care guidelines.

## Supplementary Materials

The following are available online at https://www.mdpi.com/1420-3049/24/18/3335/s1: Figure S1: AF4 is a more selective but less potent inhibitor of breast cancer cell growth, Figure S2: AF4 induces G_0_/G_1_ cell cycle arrest in MDA-MB-468 breast cancer cells, Figure S3: AF4 treatment causes ROS accumulation in non-malignant MCF-10A mammary epithelial cells, Figure S4: MDA-MB-231 breast cancer cells are more sensitive than non-malignant MCF-10A mammary epithelial cells to oxidative stress, Figure S5: Akt inhibition causes apoptosis of MDA-MB-231 breast cancer cells.
