# Supplementary figures and images for: Apple Peel Flavonoid Fraction 4 Suppresses Breast Cancer Cell Growth by Cytostatic and Cytotoxic Mechanisms
Source: Molecules. 2019 Sep 13;24(18):3335. doi: 10.3390/molecules24183335 (PMC6766994; doi:10.3390/molecules24183335)

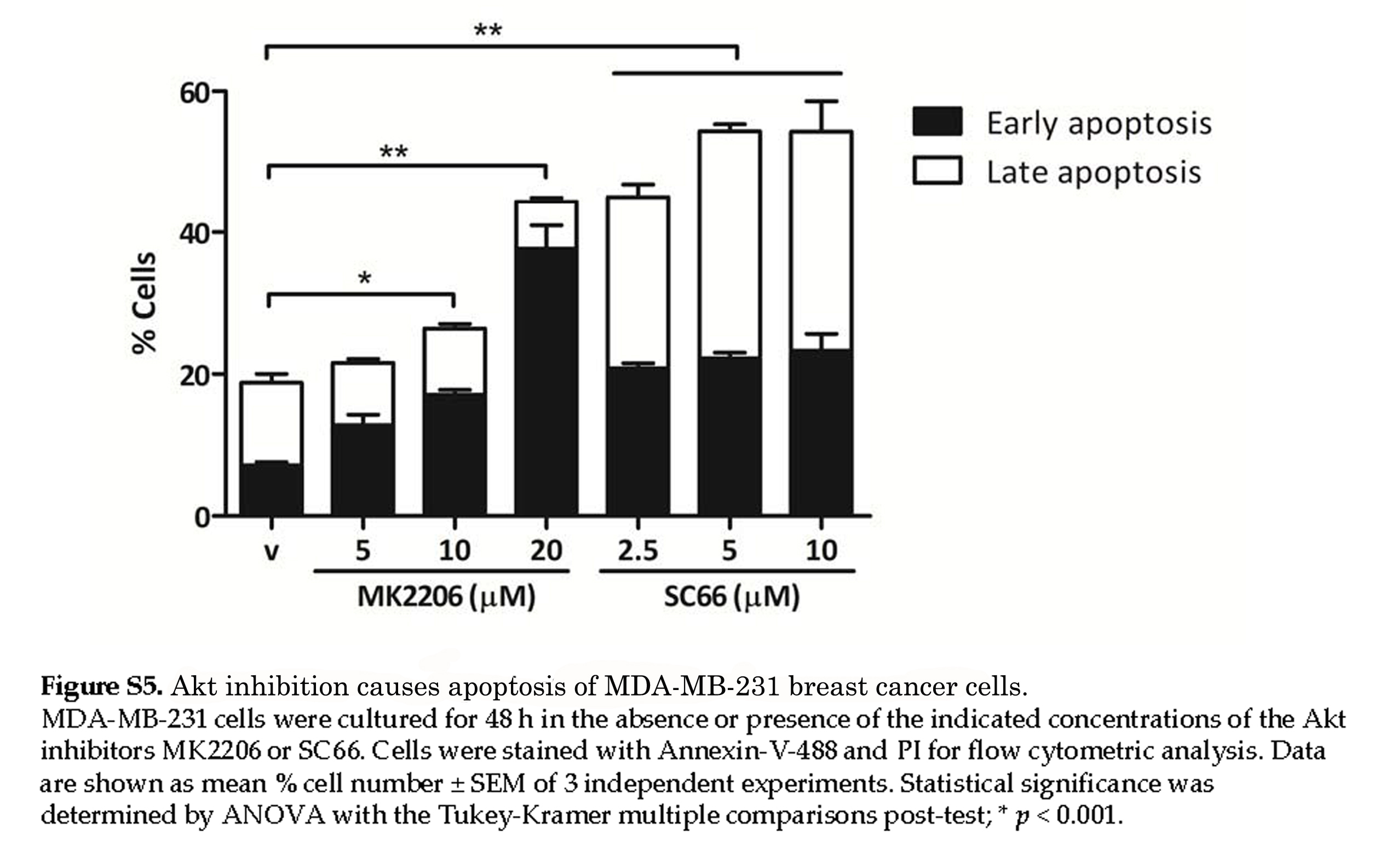

Supplement: Supplementary file 1 [file molecules-24-03335-s001.zip › Figure S5.jpg]

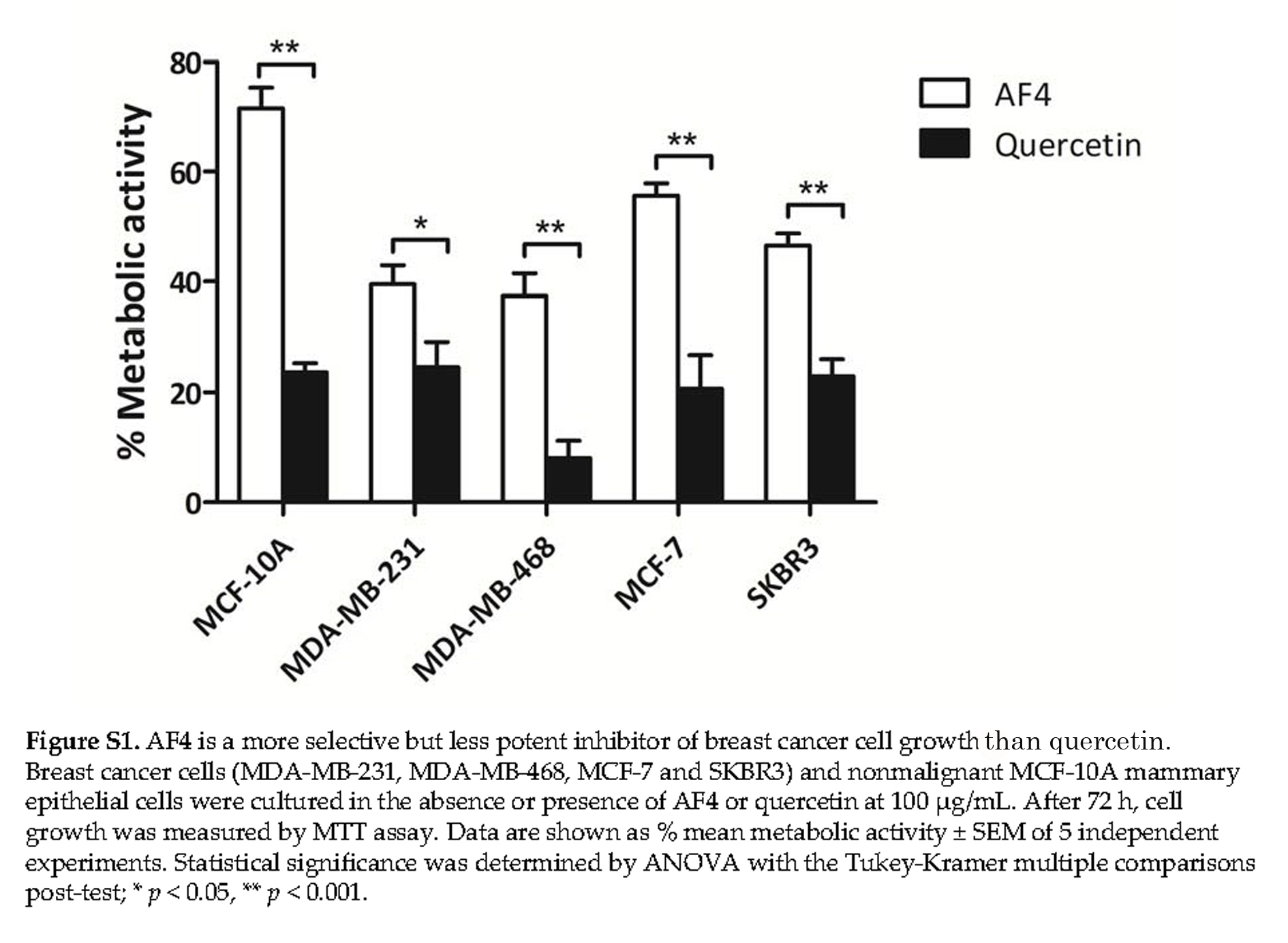

Supplement: Supplementary file 1 [file molecules-24-03335-s001.zip › Figure S1.jpg]

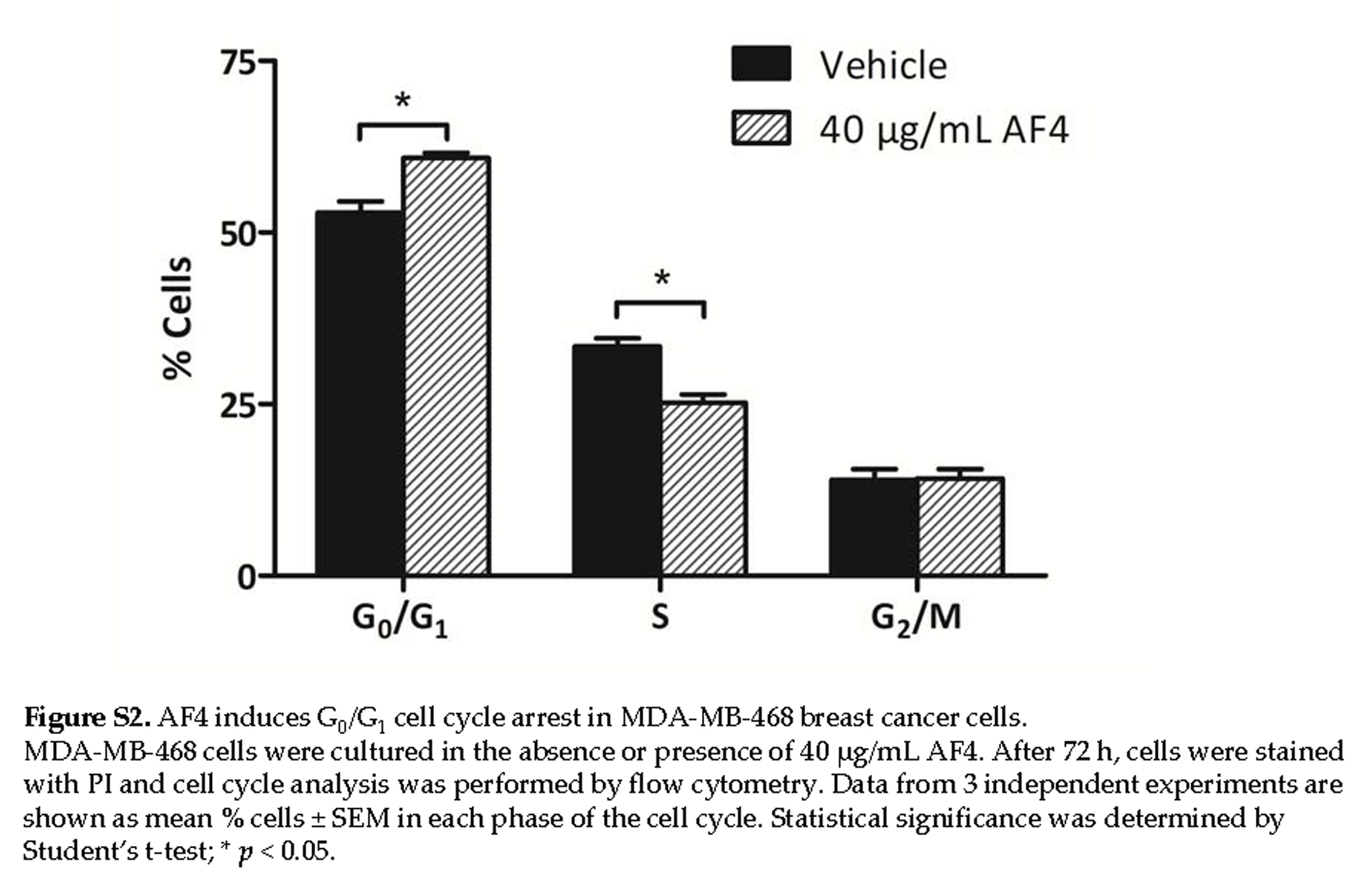

Supplement: Supplementary file 1 [file molecules-24-03335-s001.zip › Figure S2.jpg]

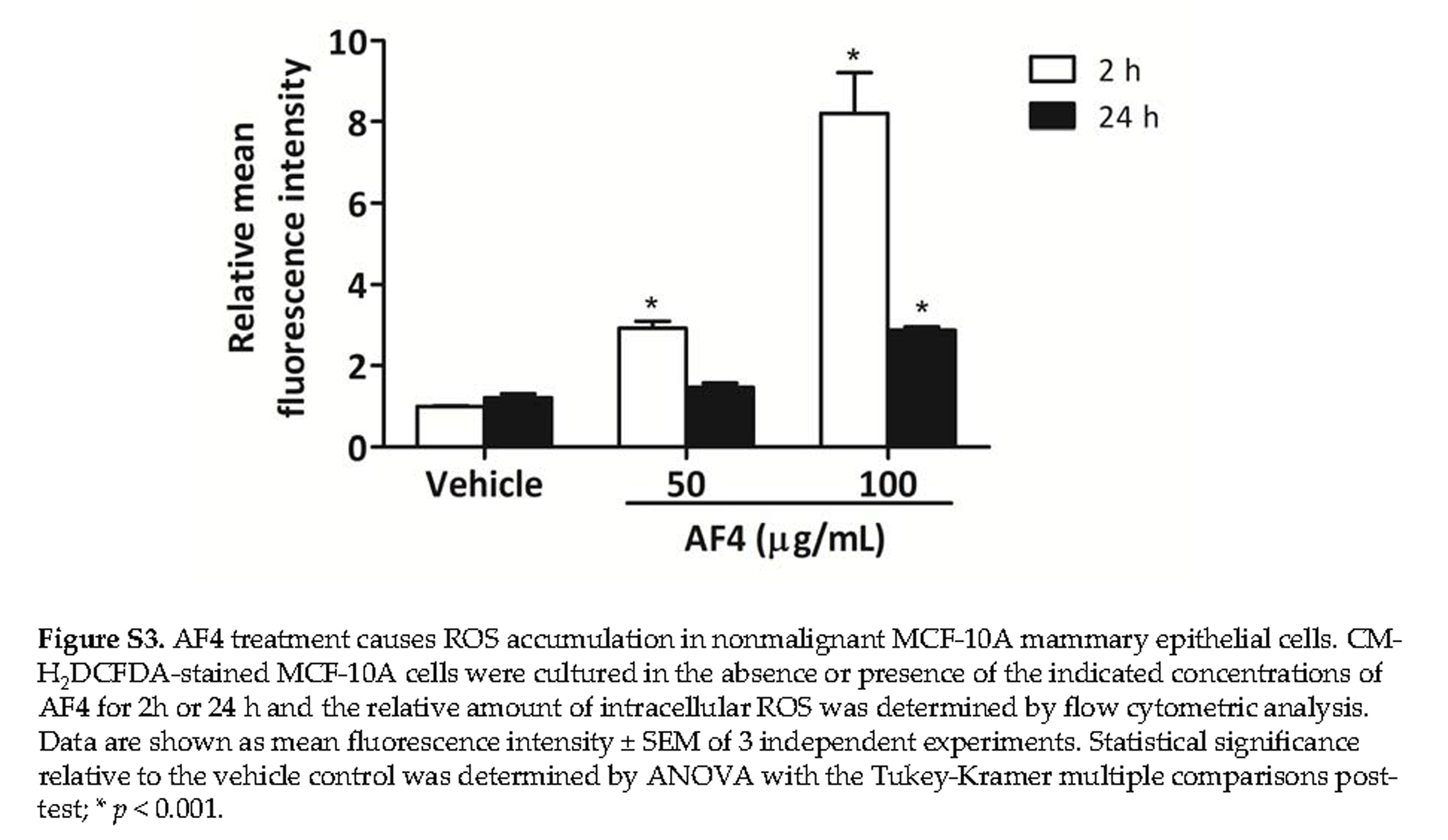

Supplement: Supplementary file 1 [file molecules-24-03335-s001.zip › Figure S3.jpg]

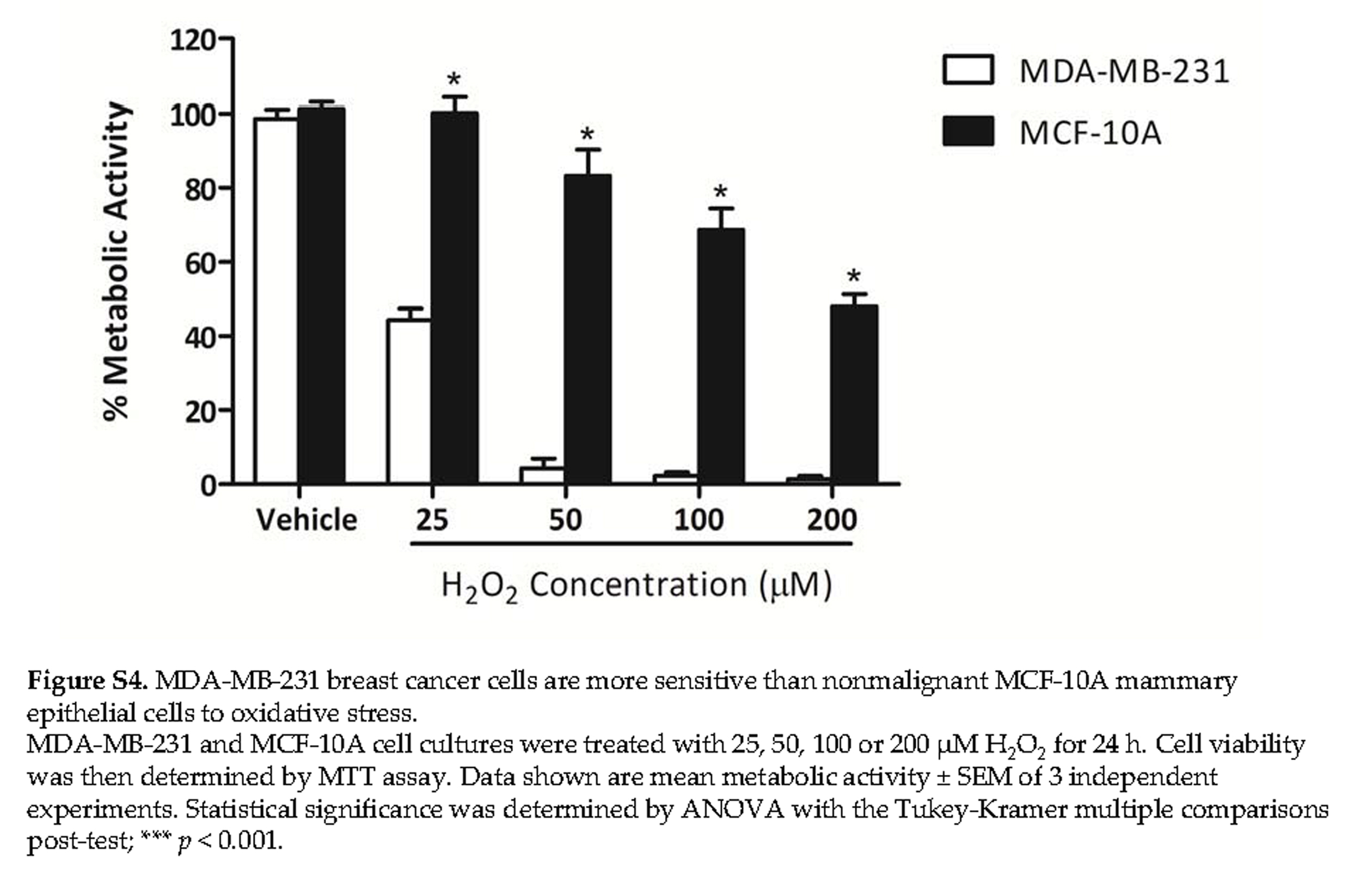

Supplement: Supplementary file 1 [file molecules-24-03335-s001.zip › Figure S4.jpg]
